# Assessing Knowledge and Awareness of Occupational Hazards and Preventive Measures among Dentists and Final-year Students in Two Dentistry Hospitals, Kabul, Afghanistan

**DOI:** 10.12688/f1000research.166053.1

**Published:** 2025-07-01

**Authors:** Fariha Kamal, Abdurrahman Anwari, Farahnaz Ghazanfari, Ahmad Anas Khairzad, Nematullah Sharifi, Fariha Omarzad

**Affiliations:** 1 Oral Medicine, Kabul University of Medical Sciences, Kabul, Kabul, 1001, Afghanistan; 2 Operative Dentistry and Endodontics, Kabul University of Medical Sciences, Kabul, Kabul, 1001, Afghanistan; 3 Dentistry Faculty, Kabul University of Medical Sciences, Kabul, Kabul, 1001, Afghanistan

**Keywords:** Occupational Hazards, Awareness, Dentistry, Preventive Measures

## Abstract

**Introduction:**

This study aimed to evaluate the knowledge and awareness of occupational hazards and preventive measures among dentists and final-year students in two dentistry hospitals in Kabul, Afghanistan.

**Methods:**

A cross-sectional descriptive study design was used in this research, which was distributed to 198 dental professionals, with Part A dealing with sociodemographic data, Part B focusing on awareness of occupational hazards, and Part C on preventive measures. Responses were analyzed to assess the level of awareness using a five-point scale. The data were also compared using Mann-Whitney U tests to examine the differences between male and female respondents and between final-year dental students and practicing dentists.

**Results:**

The overall mean proportion of correct answers was 0.71 (71%), indicating moderately high awareness. Part B had a mean of 0.73 (high), and Part C had a mean of 0.69 (medium). High awareness was found in areas such as radiation safety (93.4%), infection control (97.5%) and ergonomics (85.9%). However, gaps were identified in knowledge of lead protective gowns (15.2%) and the inverse square law (8.1%). In Part C, high awareness was noted regarding safety guidelines for electrical devices (85.9%) and protective eyewear (93.4%), but low awareness was found regarding safety boxes for sharp instruments (19.7%) and the scoop technique (31.8%). Mann-Whitney U tests revealed significant differences between male and female respondents (p < 0.001), with females showing higher awareness, although the effect size was small (r = -0.247). No significant difference was observed between the final-year students and practicing dentists (p = 0.575).

**Conclusions:**

Although dental professionals exhibit a relatively high level of awareness regarding occupational hazards and preventive measures, specific knowledge gaps persist. Addressing these deficiencies through structured educational interventions and continuous professional training is essential to enhance the occupational safety and improve the overall well-being of dental practitioners.

## Introduction

The high incidence of workplace safety hazards is a worldwide phenomenon that is particularly significant in developing countries. Working in a dangerous environment can have a negative impact on many employees’ physical and mental health, which can impact their households and close social networks. An occupational hazard is an illness or injury brought on by one’s job or the environment in which one works. ^1– 3^ The recognition of occupational risks dates back to the 18th century, when Bernardino Ramazzini, known as the ‘Father of Occupational Medicine’, first described the connection between occupation and the dynamics of health and disease. Over time, this understanding has expanded, leading to an increased awareness of various hazards in different professions, including dentistry. ^4– 8^

Occupational hazards in dentistry encompass a range of risks faced by dental professionals, including noise pollution, psychological stress, chemical exposure, infections, and musculoskeletal disorders. These hazards can significantly affect the health and well-being of dental practitioners, necessitating awareness and preventive measures to mitigate their effects. ^9, 10^ Physical and mechanical threats in dentistry include projectiles that cause eye injuries, cutting objects, and puncture wounds from needles or other sharp instruments. These injuries can also lead to the transmission of infectious diseases. Additionally, harmful radiation can damage various body cells, and prolonged exposure to noise and vibration from high-speed and low-speed handpieces, high-volume suction, and ultrasonic instruments can contribute to hearing issues. Musculoskeletal problems, such as wrist pain, lower back pain, and neck discomfort, often arise from repetitive motions and maintenance of fixed working positions. Chemical hazards include inorganic substances such as mercury, organic solvents, resins, and gases, as well as caustic chemicals such as formaldehyde. Allergic reactions such as contact dermatitis from latex gloves are also a concern. Biological hazards, including allergens, infections, toxicity of dental materials, and the risk of cross-contamination, pose additional threats. Lastly, psychological risks, such as stress, excessive workload, job dissatisfaction, professional burnout, and medicolegal concerns can significantly impact mental health. ^11, 12^

The prevalence of occupational hazards in dentistry is a significant concern, studies report an alarming prevalence of musculoskeletal disorders among dentists, and ranging from 68% to 100%, with common issues including back pain (29% to 94.6%) and neck pain (26% to 92%). ^13^ Exposure to hazardous materials such as mercury and the risk of cross infection are notable with, 44% of dental students reporting mercury toxicity and 40.4% concerned about aerosol infections. ^14^ Stress is prevalent, with 63.5% of dental professionals citing it as a major concern, often linked to patient interaction and financial pressures. ^15^

Awareness of occupational hazards in dentistry is crucial to ensure the safety of dental professionals. Various studies have highlighted the prevalence of different hazards including noise pollution, sharp injuries, chemical exposure, and blood-borne infections. Despite some awareness, a significant gap remains in the knowledge and preventive measures among dental practitioners and students. A study found that 85% of dentists perceived noise as an occupational hazard, yet only 57.1% were aware of permissible noise levels. ^16^ In a recent study conducted in Saudi Arabia, awareness of sharp injuries was satisfactory among 64.3% of dental students, but 31.3% reported exposure in the past two years. ^17^ Similar findings were also observed in another study, which demonstrated approximately 48.12% of dentists in Korea had a serious perception of occupational hazards, with 77.3% experiencing them. ^18^ Likewise, awareness of bloodborne pathogens and post exposure protocols was low in a recent study, with only 18% correctly following treatment guidelines after exposure. ^19^ Understanding these work-related threats is essential to raise awareness among dentists. ^20^ Effective safety practices in dental workplaces include the establishment of an infection prevention program, proper maintenance of work areas, implementation of education and training on physical hazards, and the correct use of personal protective equipment. ^21^

Although awareness of occupational hazards is improving, many dental professionals still lack comprehensive knowledge and adherence to safety protocols. Reviewing the literature, we found that in the context of Afghanistan, only one study has been conducted on occupational hazards in Afghanistan, which primarily focused on the occurrence and types of these hazards. ^22^ However, it did not address the knowledge and awareness of individuals regarding this topic, and no research has been conducted in this regard. In light of these considerations, the objective of the current study was to assess the level of awareness regarding occupational hazards and the preventive measures taken by both dentists and final-year students at the Stomatology Teaching Hospital and the National Stomatology Curative and Specialized Hospital in Kabul, Afghanistan.

## Material and Methods

This study was a cross-sectional, survey-based research conducted at the Stomatology Teaching Hospital and National Stomatology Curative and Specialized Hospital, Kabul, Afghanistan. These two hospitals are major dentistry healthcare facilities in Kabul City. Convenience sampling was used to recruit participants. The study included all dentists and final-year dental students from the aforementioned hospitals, who agreed to participate. In total, 198 participants were included in this study. Individuals who declined to participate were excluded from this study.

A self-designed questionnaire was developed to assess the knowledge and awareness of occupational hazards and preventive measures in dentistry. It was initially written in English, reviewed, and modified based on feedback from senior dentistry instructors and then translated into Dari for participant convenience. A pilot study was conducted with ten final-year dental students to evaluate the clarity and reliability of the questionnaire. Based on feedback from the pilot study, necessary modifications were made before full-scale data collection.

The questionnaire consisted of three main sections: Part A gathered socio-demographic information (QA1-QA5), including questions related to the participants’ age, gender, education, work experience, and position within the dental field. Part B assessed knowledge and awareness regarding occupational hazards in dentistry (QB1-QB23), covering topics such as risks from dental materials, radiation exposure, sharp injuries, and infectious diseases. Part C focused on knowledge and awareness of preventive measures for occupational hazards (QC1-QC24), addressing topics such as personal protective equipment (PPE), sterilization protocols, infection control, and safe handling practices. Each question in Parts B and C offered three response options: “Yes,” “No/Not Established,” and “I Don’t Know.”

Before data collection, data collectors were comprehensively trained to guide participants in completing the questionnaire and ensuring that all the questions were answered. All potential respondents were informed about the study’s objectives, procedures, and their rights, including confidentiality and voluntary nature of their involvement. Participation was entirely voluntary and anonymous. Written informed consent was not obtained due to the minimal-risk nature of the study, which involved non-selective data collection through anonymous, self-administered questionnaires. Requiring written consent would have introduced unnecessary administrative burden, particularly in clinical and educational settings. Therefore, verbal informed consent was obtained, and the completion and return of the questionnaire was considered as implied consent. This approach was reviewed and approved by the research committee of the Kabul University of Medical Sciences. As all participants were adults (final-year students and dentists), no minors were involved, thus no parental consent or assent was required.

The collected data were entered, systematically coded, cleaned, and analyzed using SPSS version 25. To ensure data quality, a random selection of questionnaires was compared to the data entered at the end. Correct answers to each question were assigned 1 point, while incorrect answers and ‘I do not know’ responses were assigned 0 points. The proportion of correct answers was calculated for each participant separately for Parts B and Part C. The average proportion of correct responses was then calculated across all participants for Part B (occupational hazards knowledge), Part C (preventive measures knowledge), and overall (Parts B and C combined). Awareness levels were categorized as follows: 0.00 – 0.20 (Very Low), 0.21 – 0.40 (Low), 0.41 – 0.70 (Moderate), 0.71 – 0.90 (High), and 0.91 – 1.00 (Very High).

The Mann-Whitney U test was used to compare awareness levels between male and female respondents and between final-year dental students and practicing dentists. The effect size was measured using rank-biserial correlation (r). The study was approved by the Research Committee of the Kabul University of Medical Sciences on 24/4/1403 (14/7/2024), protocol 6, agenda no. 3.

## Results

A total of 198 dental professionals participated in this study, including 106 males (53.5%) and 92 females (46.5%). The majority of respondents (90.9%) were aged between 21 and 30 years. Regarding their professional status, 131 (66.2%) were final-year dental students and 67 (33.8%) were dental practitioners. Most participants (80.8%) were affiliated with the Stomatology Teaching Hospital, whereas others were from private dental clinics or academic settings ( Table 1).

**Table 1.** Demographic distribution of respondents.

Category	Subcategory	Frequency	Percentage
**Gender**	Male	106	53.5%
	female	92	46.5%
	**Total**	**198**	**100%**
**Age group**	21-30	180	90.9%
	31-40	9	4.5%
	41-50	4	2.0%
	More than 50	5	2.5%
	**Total**	**198**	**99.9%**
**Clinical Profession**	Final year students	131	66.2%
	Dentist	67	33.8%
	**Total**	**198**	**100%**
**Hospital**	Stomatology Teaching Hospital	160	80.8%
	National Stomatology Curative and Specialty Hospital	38	19.2%
	**Total**	**198**	**100%**

Awareness of occupational hazards among participants was assessed using a set of 14 questions. The mean proportion of correct responses was 0.73, which reflected a high level of general awareness. Several questions demonstrated a particularly high awareness. For instance, 97% of respondents correctly recognized that high-intensity light used during dental procedures can cause eye damage, and 93.4% acknowledged the harmful effects of X-ray radiation exposure. Furthermore, 97.5% were aware that diseases such as Herpes Simplex Virus (HSV), Hepatitis B (HBV), HIV, and Tuberculosis (TB) can be transmitted during dental procedures. However, certain areas revealed notable gaps in their knowledge. Only 14% of participants correctly identified carpal tunnel syndrome as a common occupational hazard in dentistry. Additionally, a significant proportion (62.1%) mistakenly believed that mercury in dental amalgam was carcinogenic. Awareness of other hazards, such as the effects of poor posture on musculoskeletal health and noise-induced hearing loss, showed moderate levels of understanding, with correct response rates ranging from 50% to 70% ( Table 2).

**Table 2.** Findings from the analysis of responses to questions regarding awareness of occupational hazards (Part B of the questionnaire).

No.	Questions	Responses				Correct Answer (Proportion, category)
		Yes (Freq, %)	No/Not well established (Freq, %)	I don’t know (Freq, %)	Total (Freq, %)	
QB1	Can electrical devices in dentistry cause injuries like; burns or short circuits?	62, 31.3%	123, 62.1%	13, 6.6%	198, 100%	0.31, Low
QB2	Can low intensity light at work place cause eye pain, eye fatigue or headache?	182, 91.9%	14, 7.1%	2, 1.0%	198, 100%	0.92, Very High
QB3	Can high intensity light at work place cause eye pain, eye fatigue or headache?	192, 97.0%	4, 2.0%	2, 1.0%	198, 100%	0.97, Very High
QB4	Can light emitted from devices such as light curing devices, PC monitors, or lasers induce conjunctivitis or keratitis?	151, 76.3%	17, 8.6%	30, 15.2%	198, 100%	0.76, High
QB5	Is it correct that, high speed handpieces, air compressors, suction machines or scalers may cause hearing loss?	136, 68.7%	43, 21.7%	19, 9.6%	198, 100%	0.69, Moderate
QB6	Is there any relationship between intensity, frequency and, duration of exposure the aforementioned points and hearing loss?	170, 85.9%	13, 6.6%	15, 7.6%	198, 100%	0.86, High
QB7	Does exposure to X-ray radiation without any further protective measurement cause physical or genetical harm?	185, 93.4%	8, 4.0%	5, 2.5%	198, 100%	0.93, Very High
QB8	Does exposure to X-rays without proper protective measure gradually threatens life?	177, 89.4%	15, 7.6%	6, 3.0%	198, 100%	0.89, High
QB9	Are HSV, HBs, HIV, HCV, and TB the most worrisome infectious agents among dental practitioners?	193, 97.5%	4, 2.0%	1, 0.5%	198, 100%	0.97, Very High
QB10	In addition to bacteria, virus, and fungi, are prions also considered a biological threat?	125, 63.1%	8, 4.0%	65, 32.8%	198, 100%	0.63, Moderate
QB11	Are saliva and gingival-crevicular fluid a main source of infection?	181, 91.4%	10, 5.1%	7, 3.5%	198, 100%	0.91, Very High
QB12	Do aerosols produced during dental procedures play role in infection transmission?	151, 76.3%	21, 10.6%	26, 13.1%	198, 100%	0.76, High
QB13	Is it true that high speed handpiece produce aerosol?	152, 76.8%	24, 12.1%	22, 11.1%	198, 100%	0.77, High
QB14	Does ultrasonic scalers produce highest number of aerosols?	123, 62.1%	33, 16.7%	42, 21.2%	198, 100%	0.62, Moderate
QB15	Are injuries with sharp objects like needle and scalpel very dangerous in terms of cross-contamination?	190, 96.0%	5, 2.5%	3, 1.5%	198, 100%	0.96, Very High
QB16	Is mercury in dental amalgam a risk factor for oral cancer?	123, 62.1%	43, 21.7%	32, 16.2%	198, 100%	0.22, Low
QB17	Does continuous exposure/contact to methyl-acrylate may cause allergic reaction?	141, 71.2%	7, 3.5%	50, 25.3%	198, 100%	0.71, High
QB18	Is it true that, allergy to latex gloves is relatively common?	121 61.1%	66, 33.3%	11, 5.6%	198, 100%	0.61, Moderate
QB19	Are musculoskeletal disorders common among dental professionals?	170, 85.9%	22, 11.1%	6, 3.0%	198, 100%	0.86, High
QB20	Is carpal tunnel syndrome a common type of musculoskeletal disorder in dentistry?	135, 68.2%	28, 14.1%	35, 17.7%	198, 100%	0.14, Very Low
QB21	Is it true that, dental profession is associated with high level of mental stress?	141, 71.2%	43, 21.7%	14, 7.1%	198, 100%	0.71, High
QB22	Are lack of social activities or a decline in time spent with family, one of the major causes of stress among Dental professionals?	149, 75.3%	38, 19.2%	11, 5.6%	198, 100%	0.75, High
QB23	Are dental office wastes considered hazardous?	174, 87.9%	21, 10.6%	3, 1.5%	198, 100%	0.88, High
			**Mean Proportion of Correct Answers**			**0.73, High**

The section on preventive strategies consisted of 11 questions, with participants achieving a mean correct response rate of 0.69, indicating a slightly lower awareness than the hazard-focused questions. High levels of awareness were evident in some areas: 93.9% of participants recognized the importance of taking a detailed medical history to prevent adverse reactions, and 93.4% emphasized the need for protective eyewear during dental procedures. Moreover, 90.9% believed that managing stress by spending time with family was an effective coping strategy for psychological well-being. Nonetheless, other responses indicated critical gaps in knowledge. Only 8.1% correctly understood the inverse square law in radiation protection and 19.7% were aware of the correct use of safety boxes for disposing of sharp instruments. Similarly, only 31.8% answered correctly regarding the use of the scoop technique to prevent needle stick injury. While awareness of the benefits of proper lighting, regular breaks, and ergonomic seating was moderate, some areas related to safe handling and disposal practices were clearly under recognized ( Table 3).

**Table 3.** Findings from the analysis of questions focused on awareness regarding preventive measures of occupational hazards in dentistry (Part C of the Questionnaire).

No.	Questions	Responses				Correct Answer (Proportion, Category)
		Yes (Freq, %)	No/Not well established (Freq, %)	I don’t know (Freq, %)	Total (Freq, %)	
QC1	Can using guideline/manual for electrical devices reduce the chance of injuries caused by them?	170, 85.9%	16, 8.1%	12, 6.1%	198, 100%	0.86, High
QC2	Is it right that the use of standard light on dental units may prevent light-induced eye injury?	147, 74.2%	41, 20.7%	10, 5.1%	198, 100%	0.74, High
QC3	Can appropriate protective eye-wears provide protection against light emitted from light curing device, laser machine or pc monitors?	185, 93.4%	9, 4.5%	4, 2.0%	198, 100%	0.93, Very High
QC4	Is it correct that, dentists should adhere to radiation protection protocols while taking periapical x-rays?	168, 84.8%	22, 11.1%	8, 4.0%	198, 100%	0.85, High
QC5	Is it mandatory for physicians to wear lead protective gowns when taking periapical radiographs?	162, 81.8%	30, 15.2%	6, 3.0%	198, 100%	0.15, Very Low
QC6	Is the inverse square law (law of distance and position) utilized for the protection of dental professionals from X-rays?	16, 8.1%	145, 73.2%	37, 18.7%	198, 100%	0.08, Very Low
QC7	Is dosimetry good for monitoring x ray radiation exposure?	150, 75.8%	8, 4.0%	40, 20.2%	198, 100%	0.76, High
QC8	Does patient’s medical history help in preventing the spread of infectious agents to healthcare workers?	186, 93.9%	9, 4.5%	3, 1.5%	198, 100%	0.94, Very High
QC9	Is it true that facial masks don’t provide full protection against aerosols?	90, 45.5%	94, 47.5%	14, 7.1%	198, 100%	0.45, Moderate
QC10	Has scoop technique been designed to reduce needle stick injuries?	63, 31.8%	25, 12.6%	110, 55.6%	198, 100%	0.32, Low
QC11	Does safety boxes reduce the incidence of injuries caused by sharp instruments?	39, 19.7 %	150, 75.8%	9, 4.5%	198, 100%	0.20, Very Low
QC12	Can cleaning contaminated equipment with ultrasonic cleaning device in contrast to manual cleaning decrease the risk of sharp instrument injury?	125, 63.1%	54, 27.3%	19, 9.6%	198, 100%	0.63, Moderate
QC13	Is it necessary for dental professionals to take the Hepatitis-B vaccine?	156, 78.8%	16, 8.1%	26, 13.1%	198, 100%	0.79, High
QC14	After completion of hepatitis-B vaccine doses, is booster doses necessary for healthcare workers?	103, 52.0%	34, 17.2%	61, 30.8%	198, 100%	0.52, Moderate
QC15	Can pre-capsulated amalgam reduce the chance of injuries caused by mercury?	151, 76.3%	13, 6.6%	34, 17.2%	198, 100%	0.76, High
QC16	Is use of amalgamator safer than mixing amalgam in mortar and pestle?	163, 82.3%	15, 7.6%	20, 10.1%	198, 100%	0.82, High
QC17	Can wearing personal protective equipment reduce the chance of allergic reactions?	168, 84.8%	14, 7.1%	16, 8.1%	198, 100%	0.85, High
QC18	Does considering a balanced body position reduce the chance of musculoskeletal disorders?	158, 79.8%	14, 7.1%	26, 13.1%	198, 100%	0.80, High
QC19	Does taking short breaks during work reduce the chance of musculoskeletal disorders?	177, 89.4%	9, 4.5%	12, 6.1%	198, 100%	0.89, High
QC20	Does regular physical exercise help prevent musculoskeletal disorders among dental professionals?	163, 82.3%	24, 12.1%	11, 5.6%	198, 100%	0.82, High
QC21	Does dental microscope help dentists to maintain a balanced body position?	168, 84.8%	11, 5.6%	19, 9.6%	198, 100%	0.85, High
QC22	Does spending more time with friends and family help in reduction of stress levels among healthcare workers?	180, 90.9%	6, 3.0%	12, 6.1%	198, 100%	0.91, Very High
QC23	Is engaging in activities like yoga, drawing, or painting beneficial in reducing stress levels among dental professionals?	159, 80.3%	25, 12.6%	14, 7.1%	198, 100%	0.80, High
QC24	Does the waste disposal of dental healthcare facilities require standard protocols?	177, 89.4%	13, 6.6%	8, 4.0	198, 100%	0.89, High
		**Mean Proportion of Correct Answers**				**0.69, Medium**

The combined mean proportion of correct responses across both sections (Parts B and C) was 0.71 ( Table 4), reflecting an overall high level of awareness among participants. However, the variability in responses suggests that while some topics are well understood, others, particularly those involving technical or procedural details, require further emphasis in training and continuing education programs.

**Table 4.** Combined proportion of correct answers.

Mean Proportion of Correct Answers for Part B	0.73, High
Mean Proportion of Correct Answers for Part C	0.69, Medium
**Combined Proportion of Correct Answers**	**0.71, High**

To determine differences in awareness by gender and professional status, the Mann-Whitney U test was used. The results revealed a statistically significant difference in awareness scores between the male and female participants, with females showing significantly higher awareness levels (p = 0.000) ( Table 5). In contrast, no statistically significant difference was found between the final-year students and dental practitioners (p = 0.575), suggesting that both groups had comparable levels of knowledge regarding occupational hazards and preventive measures ( Table 6).

**Table 5.** Mann-Whitney U test results comparing male and female awareness levels regarding occupational hazards and their preventive measures.

Group Comparison	N (Males)	N (Females)	U	Z	p-value	Mean Rank (Males)	Mean Rank (Females)
Males vs. Females	106	92	3480.00	-3.481	.000	86.33	114.67

**Table 6.** Mann-Whitney U test results comparing final year students and dentists’ awareness levels regarding occupational hazards and their preventive measures.

Group Comparison	N (Final Year Students)	N (Dentists)	U	Z	p-value	Mean Rank (Final Year Students)	Mean Rank (Dentists)
Final Year Students vs. Dentists	131	67	4175.00	-0.561	.575	101.13	96.31

## Discussion

This study assessed the knowledge and awareness of occupational hazards and their preventive measures among dentists and final-year dental students at two dentistry hospitals in Kabul, Afghanistan. The overall awareness level was high (0.73) for hazards, and medium (0.69) for preventive measures, resulting in a combined awareness level of 0.71. However, specific knowledge gaps have been identified, particularly regarding the risks from electrical devices, mercury exposure, and certain musculoskeletal conditions. Notably, female participants had significantly higher awareness than male participants, while no significant difference was observed between final-year students and practicing dentists.

To the best of our knowledge, no study has used a specific methodology to assess occupational hazard awareness among dental professionals in Afghanistan. However, the findings from existing studies offer valuable insights and provide a context for understanding the results of our study.

Studies involving dental students have reported high levels of awareness regarding needlestick injuries (NSI), with 89.23% of students demonstrating knowledge of NSI and their management in one study. ^23^ Similarly, 89% of students in another study were aware of post exposure prophylaxis for accidental NSI. In our study, a high level of awareness was observed in response to QB15, where 96.0% of respondents correctly identified the dangers of cross-contamination from sharp objects like needles and scalpels, resulting in a proportion of 0.96. This finding suggests that awareness of NSI is strong among dental professionals in Afghanistan, similar to findings in other regions.

One study found that 98% of dental practitioners were aware of the risk of noise-induced hearing loss (NIHL), in contrast to only 34% of dental students. ^24^ In our study, when exploring the potential for devices such as high-speed handpieces and suction machines to cause hearing loss (QB5), the awareness level was moderate (0.69). While this is lower than the awareness found in practitioners in other studies, it highlights an important area where further education can improve understanding, especially among dental students and new professionals.

Another study found that general dentists were more likely to recognize X-rays as a risk factor than specialists. ^25^ Our study found that awareness of the harmful effects of X-ray radiation without protective measures (QB7) was very high (0.93). However, awareness slightly decreased in QB8, which assessed knowledge of the life-threatening risks posed by prolonged X-ray exposure, resulting in a proportion of 0.89, which is still classified as high. This decrease may reflect the complexity of understanding long-term risks even among those who are aware of immediate hazards.

A study assessing ergonomic awareness has generally found that women are more likely to recognize their importance than men. ^26^ Similarly, one study reported that 75.8% of female dentists had an idea of what the term ergonomics meant compared to 52.6% of male dentists, with a statistically significant difference (p < 0.05). ^27^ Our study also found a gender difference, with female participants demonstrating a higher overall awareness. This suggests that women may be more attuned to ergonomics, possibly because of both their education and the demands of dental practice.

A study assessing basic knowledge of theoretical ergonomics, the ISO 11226 standard, and ways to improve undergraduate ergonomics training found that most students had an average level of knowledge of dental ergonomic principles. ^28^ In our study, the proportion of correct responses to three key questions—whether a balanced body position (0.80), taking short breaks (0.89), and regular physical exercise (0.82) help prevent musculoskeletal disorders—was categorized as high. While the data may not be directly comparable due to differences in questioning, our findings suggest a strong awareness of practical ergonomic principles among the participants.

While no significant difference was found in awareness levels between final-year dental students and practicing dentists, this result could be partly explained by the composition of the sample. With 131 final-year students out of 198 total participants, a large proportion of students may have introduced a bias, as these individuals are currently undergoing extensive training in clinical settings, likely acquiring knowledge about occupational hazards and preventive measures at a similar rate as practicing professionals. As a result, both groups may have had relatively similar exposure to occupational health and safety education, which could account for the lack of a significant difference.

Moreover, the absence of a substantial difference could suggest that formal education on occupational hazards provided during the students’ academic curriculum might be sufficient to ensure an awareness level comparable to that of practicing dentists. It is also possible that further practical experience in the field might not drastically change awareness if foundational knowledge imparted during education is strong.

The consequences of occupational hazards extend beyond physical injury. Lack of awareness can lead to psychological issues, such as anxiety, depression, and post-traumatic stress disorder (PTSD). When healthcare workers experience repeated exposure to hazardous situations without proper knowledge and support, their mental health deteriorates, leading to burnout and decreased job satisfaction. ^1, 29^ Inadequate awareness can not only affect the overall productivity of healthcare workers, but also compromises patient safety and outcomes. ^30, 31^

Furthermore, inadequate awareness has a substantial economic impact. Increased healthcare costs due to injuries, loss of productivity, and the need for additional training or recruitment can limit healthcare budgets. In low- and middle-income countries (LMICs), where resources are already limited, this becomes a critical issue affecting the sustainability of health services. ^31, 32^

Our study identified several clear gaps in the knowledge regarding occupational hazards and preventive measures, which may contribute to these broader consequences. Notably, there is insufficient awareness about the risks posed by electrical devices, mercury in dental amalgams, and specific musculoskeletal conditions such as carpal tunnel syndrome. Similarly, gaps were evident in the understanding of radiation safety measures, including the importance of wearing lead-protective gowns, applying the inverse square law, and the use of safety boxes for sharp instruments. Addressing these knowledge gaps is essential not only for individual safety, but also for improving mental well-being, reducing economic burdens, and enhancing overall healthcare sustainability.

These gaps highlight the need for regular and effective training on occupational hazards and preventive measures. Studies indicate that healthcare workers LMICs, including dental professionals, often lack adequate training, resulting in insufficient knowledge about critical risks, such as blood-borne pathogens and ergonomic hazards. Addressing these deficiencies requires ongoing educational intervention to improve awareness and safety practices.

Support from health organizations through counseling services and workplace health programs can play a pivotal role in enhancing awareness. Additionally, analyzing the history of workplace incidents can help identify weaknesses in safety protocols, which can then be used to tailor more effective training programs. It is critical to build and sustain a strong safety culture within dental workplaces, as this has been shown to positively influence employee behavior and adherence to safety protocols. A culture of safety reinforced through continuous education and leadership support can significantly reduce the risks associated with dental practice and improve the well-being of dental professionals.

Creating a safe work environment is essential to reduce occupational hazards. Many healthcare workers are exposed to various types of hazards, as mentioned before in this study, which can be mitigated through proper safety protocols. ^33, 34^ Challenging working conditions, such as long hours and high stress, contribute to burnout and this condition can impair their ability to recognize and respond to hazards effectively. ^30, 35^

Regular and effective training on occupational hazards and preventive measures is critical. Studies show that healthcare workers in LMICs often lack adequate training, leading to insufficient knowledge about risks, such as blood-borne pathogens and ergonomic hazards. Support from organizations through counseling services and health programs can enhance awareness, and analyzing the history of workplace incidents can help identify safety weaknesses and improve training programs. A strong safety culture within the workplace significantly impacts employee behavior and adherence to safety protocols. ^36, 37^

This study had certain limitations. The use of a self-made questionnaire may have impacted the validity and reliability of the findings, as it was not pre-validated against standardized measures. Additionally, the convenience sampling method limits the generalizability of the results, as the participants may not be fully representative of all dental professionals in Kabul. Future research should consider using a validated questionnaire and randomized sampling approach to improve the robustness of the findings.

Future research could explore intervention-based approaches, such as training programs, to assess whether targeted education might enhance awareness of lesser-known occupational hazards. Additionally, conducting a larger multicenter study involving different regions of Afghanistan could offer a more comprehensive understanding of awareness levels nationwide. By addressing these issues, future studies may contribute to further enhancing the safety and well-being of dental professionals.

## Conclusion

In conclusion, while dental professionals exhibit a relatively high level of awareness of occupational hazards and preventive measures, specific knowledge gaps persist. Addressing these deficiencies through structured educational interventions and continuous professional training is essential to enhance the occupational safety and improve the overall well-being of dental practitioners.

## Ethics approval and informed consent

The study was approved by the Research Committee of the Kabul University of Medical Sciences on 24/4/1403 (14/7/2024), protocol 6, agenda no. 3. Verbal informed consent was obtained, and the completion and return of the questionnaire was considered as implied consent.

## Data Availability

The datasets generated and analyzed during the current study are publicly available on Figshare:
▪ SPSS dataset: Anonymized raw dataset in.sav format used for statistical analysis. https://doi.org/10.6084/m9.figshare.29328428.v1 ^38^
▪ Questionnaire file: Structured blank survey questionnaire (pdf in Dari and English) used for data collection in this study. https://doi.org/10.6084/m9.figshare.29328377.v1 ^38^ Data are available under the terms of the Creative Commons Attribution 4.0 International license (CC-BY 4.0).
