# Supplementary material for: Assessing Knowledge and Awareness of Occupational Hazards and Preventive Measures among Dentists and Final-year Students in Two Dentistry Hospitals, Kabul, Afghanistan
Source: F1000Res. 2025 Sep 9;14:639. Originally published 2025 Jul 1. [Version 3] doi: 10.12688/f1000research.166053.3 (PMC12457895; doi:10.12688/f1000research.166053.3)
Supplement: Peer review history

# Reviewer report: Reviewer response for version 3

**DOI:** 10.5256/f1000research.187757.r413236

**Published:** 2025-09-23

**Authors:** Putranto Manalu

**Affiliations:** 1 Universitas Prima Indonesia, Medan, Indonesia

I have reviewed the submission and confirm that I have the necessary expertise to evaluate its scientific merit. This study addresses a crucial and relevant topic: the awareness of occupational hazards among dental professionals, including both final-year students and practicing dentists. The research is valuable as it highlights specific knowledge gaps and misconceptions that have direct implications for the health, safety, and well-being of dental practitioners. The finding of no significant difference in awareness between students and experienced dentists is particularly noteworthy and warrants further discussion.

 While the study is promising, there are several key areas that require significant revision to strengthen the methodology, deepen the analysis, and enhance the overall impact of the manuscript. The following report provides a summary of the article's findings and expands on four major points that must be addressed before the article can be considered scientifically sound for publication.

**1. Inability to Determine the Study's Response Rate**

 A significant methodological limitation is the inability to calculate the study's response rate. The authors report that 198 dental professionals participated in the survey. However, they also state that the precise number of eligible individuals who were invited but declined to participate or were absent could not be determined. Consequently, the response rate could not be calculated. This omission raises concerns regarding potential non-response bias, as the resulting sample may not be fully representative of the target population of dentists and final-year students in the selected hospitals. It is plausible that individuals with a higher pre-existing awareness of occupational hazards were more inclined to participate, potentially skewing the results toward a higher overall awareness level.

**2. Discrepancy in Awareness of Musculoskeletal Disorders (MSDs)**

 The results present a notable discrepancy regarding awareness of MSDs. Participants demonstrated high general awareness that MSDs are a common hazard in dentistry, with 85.9% correctly identifying them as such. In stark contrast, awareness of carpal tunnel syndrome as a specific and common type of MSD was exceptionally low, with only 14% of participants providing the correct answer. The manuscript's discussion section does not sufficiently explore the reasons for this significant gap between general knowledge and specific knowledge. An in-depth analysis of potential causes, such as deficiencies within the dental education curriculum regarding specific ergonomic-related conditions, would provide valuable context for this finding.

**3. Substantiation of "Correct" Answers Regarding Mercury Carcinogenicity**

 The study classifies the belief that mercury in dental amalgam is a risk factor for oral cancer as incorrect. A majority of respondents (62.1%) held this belief. Given that a significant proportion of the professional sample holds this view, the manuscript's argument would be strengthened by substantiating the "correct" answer with a citation from an authoritative body, such as the World Health Organization or a major international dental association. This would provide a firm evidentiary basis for labeling the belief as a misconception and would offer readers important context.

**4. Interpretation of No Significant Difference Between Students and Dentists**

 The finding that there was no statistically significant difference in awareness levels between final-year students and practicing dentists (p=0.575) is a key result that invites deeper interpretation. The authors propose this may be due to the large number of students in the sample or the adequacy of the formal curriculum. However, the discussion should also consider an alternative interpretation: the lack of difference could indicate a stagnation of knowledge among practicing dentists post-graduation, possibly due to insufficient or ineffective continuing professional education in occupational health and safety. Exploring this possibility would lend greater weight to the study's conclusion that continuous training is essential for enhancing and maintaining awareness among dental practitioners.

Is the work clearly and accurately presented and does it cite the current literature?

Yes

If applicable, is the statistical analysis and its interpretation appropriate?

Yes

Are all the source data underlying the results available to ensure full reproducibility?

Yes

Is the study design appropriate and is the work technically sound?

Yes

Are the conclusions drawn adequately supported by the results?

Yes

Are sufficient details of methods and analysis provided to allow replication by others?

Yes

Reviewer Expertise:

Occupational and Safety Health, Ergonomic

I confirm that I have read this submission and believe that I have an appropriate level of expertise to confirm that it is of an acceptable scientific standard, however I have significant reservations, as outlined above.

# Reviewer report: Reviewer response for version 2

**DOI:** 10.5256/f1000research.186839.r409319

**Published:** 2025-09-03

**Authors:** Wita Anggraini

**Affiliations:** 1 Universitas Trisakti, Jakarta, Indonesia

Congratulations to the authors, you have revised very well, so that your manuscript is more informative and the writing is better.

Is the work clearly and accurately presented and does it cite the current literature?

Partly

If applicable, is the statistical analysis and its interpretation appropriate?

Yes

Are all the source data underlying the results available to ensure full reproducibility?

Partly

Is the study design appropriate and is the work technically sound?

Yes

Are the conclusions drawn adequately supported by the results?

Partly

Are sufficient details of methods and analysis provided to allow replication by others?

Yes

Reviewer Expertise:

Human Anatomy, Ergonomic Dentistry,  Anthropometry and Periodontology

I confirm that I have read this submission and believe that I have an appropriate level of expertise to confirm that it is of an acceptable scientific standard.

# Reviewer report: Reviewer response for version 2

**DOI:** 10.5256/f1000research.186839.r404361

**Published:** 2025-09-01

**Authors:** Nataša Trifunović

**Affiliations:** 1 University of Sarajevo, Sarajevo, Bosnia and Herzegovina

Since changes have been made to the manuscript, I will refer to Version 2 with modifications in the Results and Discussion sections. My comment concerns the Methodology section – it is necessary to clearly and more precisely state the exclusion criteria for the study (e.g., exclusion criteria may include dentists and final-year students who, at the time of the study, were not present at work/university due to sick leave, annual leave, maternity leave, or extended medical leave). Please ensure that both the inclusion and exclusion criteria are explicitly stated in this study.

 In the introductory part of the Results section, it is important to report the number of final-year students and dentists in the region where the study was conducted, the total number of participants invited to take part, and the number who declined participation, in order to provide the response rate.

Is the work clearly and accurately presented and does it cite the current literature?

Yes

If applicable, is the statistical analysis and its interpretation appropriate?

Yes

Are all the source data underlying the results available to ensure full reproducibility?

Yes

Is the study design appropriate and is the work technically sound?

Yes

Are the conclusions drawn adequately supported by the results?

Yes

Are sufficient details of methods and analysis provided to allow replication by others?

Partly

Reviewer Expertise:

NA

I confirm that I have read this submission and believe that I have an appropriate level of expertise to confirm that it is of an acceptable scientific standard, however I have significant reservations, as outlined above.

# Response

**Published:** 2025-09-04

**Authors:** Fariha Kamal

**Affiliations:** Oral Medicine, Kabul Universwity of Medical Sciences, Kabul, Kabul, Afghanistan

We would like to thank the reviewer for their valuable comments and suggestions, which helped us improve the clarity of the manuscript.

**Comment 1:** Please state the exclusion criteria more clearly.

**Response:** Thank you for this valuable suggestion. We have revised the *Methods* section to explicitly include both inclusion and exclusion criteria. Exclusion criteria now specify individuals who declined participation, gave incomplete responses, or were absent due to sick leave, annual leave, maternity leave, or extended medical leave.

**Comment 2:** Please report the total number of eligible participants, those who declined, and the response rate.

**Response:** We appreciate this comment. In the *Results* section, we now state that 198 participants (131 students and 67 dentists) completed the survey. Some individuals may have declined or been absent, but as the exact numbers were not recorded, the precise response rate could not be determined.

# Reviewer report: Reviewer response for version 1

**DOI:** 10.5256/f1000research.182890.r401176

**Published:** 2025-08-14

**Authors:** Wita Anggraini

**Affiliations:** 1 Universitas Trisakti, Jakarta, Indonesia

Review Results:

**Material and Methods**

- **Paragraph 1:** State the inclusion and exclusion criteria in this study.
- **Paragraph 3:** What is the researcher's rationale for the 'No/Not well Established’ and "I don't know" answers?. Regarding the two "No" answers above, is it possible that final-year students, or even working dentists, have never been exposed to the occupational safety culture knowledge being studied? Please explain this in the methods section.
- **Paragraph 4, in the last sentence:** As all participants were adults (final-year students and dentists), no minors were involved, thus no parental consent or assent was required.--This statement does not need to be written, because the research method has been approved by the relevant Ethics Committee.
- **Paragraph 5:** Awareness levels were categorized as follows: 0.00– 0.20 (Very Low), 0.21– 0.40 (Low), 0.41– 0.70 (Moderate), 0.71– 0.90 (High), and 0.91– 1.00 (Very High).-- This range statement requires a library source.

**Results**

- **In Tables 1 and 2:** The Yes/No/I don't know and frequency answer columns will be clearer if they are separated into 2 columns each, for example: the yes answer column and the frequency column.

**Discussion**

- In the discussion section:
  - you need to address respondents who answered  'No/Not well established’ and ‘I don't know’.  Does this impact the results of this study?
  - you need to review how the Occupational Safety and Health curriculum is delivered during education, as this relates to the knowledge gaps and low levels of awareness found in dentists and students.
- **Paragraph 6:** Much research has been done on dental ergonomic, it is necessary to explain in more depth why there are gender differences in occupational health related to ergonomics.
- **Paragraph 16:** Are the number of hours worked per day for each respondent recorded? If yes, it would be better for the author to include it, so that it is relevant to the statements in reference sources numbers 30 and 35.

**Conclusion**

 The conclusion should be made more pointed, such as the importance of knowledge and awareness of occupational hazards and their preventive measures. It is important for dentists and students to be aware of and willing to prevent various aspects of their occupational hazards, for example: noise, light, ergonomic positions, etc.

Is the work clearly and accurately presented and does it cite the current literature?

Partly

If applicable, is the statistical analysis and its interpretation appropriate?

Yes

Are all the source data underlying the results available to ensure full reproducibility?

Partly

Is the study design appropriate and is the work technically sound?

Yes

Are the conclusions drawn adequately supported by the results?

Partly

Are sufficient details of methods and analysis provided to allow replication by others?

Yes

Reviewer Expertise:

Human Anatomy, Ergonomic Dentistry,  Anthropometry and Periodontology

I confirm that I have read this submission and believe that I have an appropriate level of expertise to confirm that it is of an acceptable scientific standard, however I have significant reservations, as outlined above.

## References

[1] : World Trends in Dental Ergonomics Research: A Bibliometric Analysis. *International Journal of Environmental Research and Public Health* .2024;21(4) : 10.3390/ijerph21040493 10.3390/ijerph21040493 PMC1105063938673404

[2] : The prevalence and risk factors of musculoskeletal disorders among Indonesian dental professionals. *Frontiers in Rehabilitation Sciences* .2025;6: 10.3389/fresc.2025.1513442 10.3389/fresc.2025.1513442 PMC1186096640012958

# Response

**Published:** 2025-08-18

**Authors:** Fariha Kamal

**Affiliations:** Oral Medicine, Kabul Universwity of Medical Sciences, Kabul, Kabul, Afghanistan

**Dear Reviewer,**

 We sincerely appreciate your thorough review of our manuscript entitled *“Assessing Knowledge and Awareness of Occupational Hazards and Preventive Measures among Dentists and Final-year Students in Two Dentistry Hospitals, Kabul, Afghanistan”*. We are grateful for your constructive comments, which have helped us to improve the quality and clarity of our paper. Below, we address each of your points in detail and indicate the corresponding changes made in the revised manuscript.

**Material and Methods**

1. *Inclusion and Exclusion Criteria:* We have now added a clear statement of the inclusion and exclusion criteria in the first paragraph of the Methods section.
2. *Rationale for “No/Not well established” and “I don’t know” answers:* We have explained that these options were included to capture both lack of knowledge and lack of familiarity with occupational safety culture, which might occur even among practicing dentists in Afghanistan due to limited exposure in curricula and continuing education. This explanation has been added to the Methods section.
3. *Removal of parental consent sentence:* The statement regarding the absence of minors and parental consent has been removed, as it is unnecessary given the ethics committee approval.
4. *Categorization ranges:* We have cited a relevant source to support the categorization of awareness levels (0.00–0.20 Very Low, 0.21–0.40 Low, etc.).

**Results**

 5. We have modified Tables 1 and 2 so that “Yes/No/I don’t know” responses and their corresponding frequencies are presented in separate columns for better clarity.

**Discussion**

 6. We have discussed the possible implications of “No/Not well established” and “I don’t know” responses, including their potential effect on the interpretation of the study results.

 7. We have added a review of how Occupational Safety and Health (OSH) topics are currently delivered in dental education in Afghanistan and related this to the identified knowledge gaps.

 8. We have elaborated on gender differences in ergonomic-related occupational health, drawing from relevant literature.

 9. We have clarified that hours worked per day were recorded and have incorporated this information into the discussion to align with references #30 and #35

**Conclusion**

 10. We have revised the conclusion to make it more focused, emphasizing the importance of awareness and prevention of various occupational hazards among both dentists and students.

 We appreciate your valuable input and believe these revisions have significantly strengthened our manuscript.

**Best regards,**

 Dr. Fariha Kamal

 (On behalf of all co-authors)
